# Knowledge, Practices, and Restrictions Related to Menstruation among Young Women from Low Socioeconomic Community in Mumbai, India

**DOI:** 10.3389/fpubh.2014.00072

**Published:** 2014-07-03

**Authors:** Harshad Thakur, Annette Aronsson, Seema Bansode, Cecilia Stalsby Lundborg, Suchitra Dalvie, Elisabeth Faxelid

**Affiliations:** ^1^Centre for Public Health, Tata Institute of Social Sciences, School of Health Systems Studies, Mumbai, India; ^2^Division of Social Medicine and Global Health, Lund University, Malmö, Sweden; ^3^Department of Preventive and Social Medicine, Seth G.S. Medical College and KEM Hospital, Mumbai, India; ^4^Division of Global Health (IHCAR), Department of Public Health Sciences, Karolinska Institutet, Stockholm, Sweden; ^5^Consultant Gynaecologist, Mumbai, India

**Keywords:** menstruation, menstrual hygiene, knowledge of menstruation, problems during menstruation, menstrual practices

## Abstract

The main objective was to assess knowledge, practices, and restrictions faced by young women regarding their menstrual hygiene. The views of adult women having young daughters were also included and both views were compared. In addition, the factors influencing the menstrual hygiene practices were also studied. The study was carried out during 2008 in Mumbai, India. The mixed methods approach was followed for the data collection. Both qualitative and quantitative methods were used to collect the data. For quantitative survey, totally 192 respondents (96 adult and 96 younger women) were selected. While young women were asked about questions related to their menstruation, adult women were asked questions to find out how much they know about menstrual history of their daughters. The qualitative data helped to supplement the findings from the quantitative survey and to study the factors affecting menstrual practices in young women. The mean age at menarche reported was 13.4 years and 30–40% of young girls did not receive any information about menstruation before menarche. It is thus seen that very few young girls between the age group 15 and 24 years did receive any information before the onset of menstruation. Among those who received some information, it was not adequate enough. The source of information was also not authentic. Both young and adult women agreed on this. Due to the inadequate knowledge, there were certain unhygienic practices followed by the young girls resulting in poor menstrual hygiene. It also leads to many unnecessary restrictions on young girls and they faced many health problems and complaints, which were either ignored or managed inappropriately. The role of health sector was almost negligible from giving information to the management of health problems of these young girls. This paper reemphasizes the important, urgent, and neglected need of providing correct knowledge to the community including adolescent girls.

## Introduction

Menstruation is a physiological process, which is associated with the ability to reproduce. The name “menstruation” comes from the Latin “menses” meaning moon, with reference to the lunar month and lasting also approximately 28 days long. Its onset profoundly changes a young woman’s life.

Menstruation has always been surrounded by different perceptions throughout the world. Nowadays, there is some openness toward menstruation, but differences in attitude still persist between different populations (1). There are differences between countries, cultures, religions, and ethnics groups. In many low-income countries, women and girls are restricted in mobility and behavior during menstruation due to their “impurity” during menstruation. In many parts of the world, menstruation is still related to a number of cultural taboos as well as feelings of shame and uncleanliness. Even today menstruation is a secret of mother and daughter in many families. It is not discussed in the open.

In India, menstruation is considered a natural event, a gift from the God, and is considered necessary as it gives womanhood. Here, women’s perceptions of menstruation vary among different cultures and religions (2). There are many taboos like menstruating girl is prevented from going to temple, to cook food, to attend weddings, etc. There is limited knowledge and many misconceptions about menstruation among young women in India before and even after the menarche. This usually leads to undue fear, anxiety, and undesirable practices (3). The knowledge and practices related to menstruation are dependent on socio economic conditions as well (4).

Today, the number of women who have regular menstruation periods is increasing in developing countries including India due to later childbearing and fewer children. But many lack the economic and social conditions to manage menstruation sanitation satisfactorily. A particularly vulnerable group in this aspect is young women in poor families. Furthermore, understanding young women’s knowledge and practices related to menstruation is a central element for designing appropriate education programs.

There is very limited social and health related research on menstruation issues in low and middle income countries including India. There is also limited research on menstruation as a social and cultural phenomenon or on the technical and hygienic aspects of sanitary protection in various socioeconomic contexts. The reason might be that menarche and menstruation are considered a taboo and thus rarely discussed (5), even between mother and daughter. The reason for mother’s reluctance to discuss menstruation and related issues with their daughters can be partially related to their own lack of knowledge of the physiology of menstruation (6).

Considering the above scenario, this study was conducted among women from an urban area with low socioeconomic status in Mumbai, India. The main objective was to assess knowledge, practices, and restrictions faced by young women (especially between the age group of 15 and 24 years) regarding their menstrual hygiene. Usually for any young girl, it is expected that her mother is the primary source of information as far as menstruation is concerned. So the views of adult women having young daughters were also included and both views were compared. In addition, the factors influencing the menstrual hygiene practices were also studied mainly through qualitative methods.

## Materials and Methods

The study was carried out during January–March 2008 in The Bombay Development Department (BDD) chawls (buildings) located in Naigaon area of Parel. This is the most central part of Mumbai, India. The BDD buildings were built up during 1920–1925 period and now are in dire need of urgent repair and replacement. There are 42 buildings in this area and it represents lower-middle class population. All these buildings have 4 floors and 20 apartments per floor. An apartment in this chawl consists of one all purpose room (functions both as a living room and bedroom) and a kitchen (also serving as a dining room). Families on a floor have to share a common block of bathrooms and latrines. There is little privacy for people living in a chawl. The majority of the population in these buildings work in the mills and industries in the surrounding area.

The mixed methods approach was followed for the data collection. Both qualitative and quantitative methods were used to collect the data. The quantitative survey was used to compare the findings between young and adult women. The qualitative data helped to supplement the findings from the quantitative survey and to study the factors affecting menstrual practices in young women.

For the quantitative survey, a structured questionnaire was used that elicited information on demographic and socioeconomic background of individuals in addition to information related to the objectives. The questionnaire was modified based on experience of the pre-test conducted before the main study. The postgraduate students from the nearby medical college collected the data through personal face-to-face interviews. These interviewers were trained by the study investigators. The informed consent was taken from the each participant before the interview started. In case the young woman was <18 years, consent was requested either from a parent or from a guardian.

During the quantitative survey, 12 buildings were selected from 42 buildings by systematic random sampling. From each floor four women were included; two women with daughters between 15 and 24 years age groups (henceforth called adult women) and two young girls between the age group of 15 and 24 years (henceforth called young women). The care was taken during the selection of the adult and young women. They were selected from different apartments and did not belong to the same family. Each chawl has four floors. Thus from each chawl, eight adult and eight young women were selected. Totally 192 respondents (96 adult and 96 young women) were selected from the 12 buildings. The adult women were almost always available for interviews during the day time. It was difficult to contact few young women since most of them were students. If the selected respondents were not present during the visit, they were visited again contacted during their convenient time. While young women were asked about questions related to their menstruation, adult women were asked to find out how much they know about menstrual history of their daughters.

In addition, the focus group discussions (FGD) and key informants (KI) interviews were conducted to collect the qualitative data. A semi-structured schedule was prepared for conducting both FGD and KI interviews. Totally five FGDs were conducted in the following groups – school, college dropout adolescent girls; college going girls (19 years and above); married women (18–29 years); nursing students; and girls from junior college (15 years and above). Totally 11 KI were interviewed as follows – government medical officer, school teacher, college teacher, mother not working, peer leaders of adolescent health initiative program, Mahila Mandal (local women group) representatives, laboratory technician, elderly mother having four daughters, public health nurse, Gynecologist in government maternity center, and medical officer in private sector. All these selected participants for qualitative methods were staying and working in the same area. These groups were deliberately selected after much brain storming among the researchers. It was felt that with these groups and informants, we will be able to get the required information on the factors influencing the menstrual hygiene practices of young women.

Both qualitative and quantitative analysis involved labeling and coding all of the data in order that similarities and differences can be recognized. Statistical analysis was done using SPSS 15 and a minimum level of statistical significance was 0.05. The qualitative data analysis involved aiming to uncover and understand the big picture. It was mainly done by content analysis – a more interpretive analysis that was concerned with the response as well as what may have been inferred or implied. The aim was to make sense of the data collected and to highlight the important messages, features, or findings.

## Results

The findings from the quantitative survey are presented in tabular format. Since the qualitative methods mainly assisted in understanding and explaining the results from the quantitative survey, so the results from the qualitative methods are mentioned simultaneously.

The socioeconomic and demographic profile of the selected participants is presented in Table 1. The average family size of the families (households) of the selected household during the household survey was 4.77. The mean per capita income was Indian Rs. (INR) 2123/- per month, which is lower than average in that particular area. Average age of the young and adult women was 19.3 and 43.5 years, respectively. As expected, the young women were better educated than the adult women. Almost all adult women were housewives (94.8%), whereas the majority of the young women (75%) were students. The mean age at menarche reported was 13.4 years.

**Table 1:** **The profile of the selected participants**.

Background characteristics	Young women	Adult women	Comments
		
	*N* (%)	*N* (%)	
**EDUCATION**			
Illiterate	0 (0.0)	6 (6.3)	Chi square = 49.42, *p* < 0.001, significant
Primary (up to 4 standard)	1 (1.0)	7 (7.3)	
Secondary (up to 10 standard)	45 (46.9)	74 (77.1)	
College (up to graduation)	37 (38.5)	5 (5.2)	
Graduate	12 (12.5)	2 (2.1)	
Post graduate	0 (0.0)	0 (0.0)	
Not available	1 (1.0)	2 (2.1)	
**OCCUPATION**			
Service	11 (11.5)	3 (3.1)	Chi square = 160.06, *p* < 0.001, significant
Business	0 (0.0)	2 (2.1)	
Student	71 (74.0)	0 (0.0)	
At home (house wife)	6 (6.3)	91 (94.8)	
Unemployed	7 (7.3)	0 (0.0)	
Not available	1 (1.0)	0 (0.0)	
**RELIGION**			
Hindu	88 (91.2)	87 (90.6)	Chi square = 1.10, *p* = 0.778, not significant
Buddhist	6 (6.3)	5 (5.2)	
Muslim	1 (1.0)	3 (3.1)	
Christian	1 (1.0)	1 (1.0)	
Total	96 (100)	96 (100)	

As shown in Table 2, while 58.3% of young women stated that they were informed about menstruation before menarche, 69.8% adult women felt that their daughters had received information prior to menarche. This implies that 30–40% of young girls did not receive any information about menstruation before menarche. FGDs and KI interviews also brought forward the point that many young girls did not knew anything before the occurrence of their first menstruation and a few were very scared when it happened. The majority of the girls also viewed menstruation as natural and a sign of womanhood and something to be proud of. However, some considered it unfair that only girls had to suffer and boys do not have to face this.

**Table 2:** Perceptions of 96 adult women and 96 younger women about menstruation issues*.

Menstruation issues	Young women’s perception about herself	Adult women’s perception about her daughter	Comments
	(*n* = 96)	(*n* = 96)	
Received information about menstruation prior to menarche	56 (58.3)	67 (69.8)	Chi square = 2.74, *p* = 0.098, not significant
Complaints faced during menstruation	68 (70.8)	63 (65.6)	Chi square = 0.601, *p* = 0.438, not significant
Restrictions faced during menstruations	82 (85.4)	85 (88.5)	Chi square = 0.41, *p* = 0.520, not significant

*Values are given as number (percentage).

Table 3 presents source and adequacy of knowledge about menstruation only among the participants who had received some information before menarche (56 young women and 67 adult women). Both the groups agreed that the school teachers were the most common source of information. Young women also consider that they received information from mothers, but mothers did not think so. Physicians played very minor role in giving information. Sixty four percent of the younger women and 81.5% of adult women said that young girls did not receive any information regarding the link between menstruation and fertility. The information received was also considered inadequate both by the adult women (76.8%) and the younger women (92.3%). Inadequate information means that they had either received very little information, which is of any practical value or they might have been misinformed depending upon the source of information. Qualitative methods revealed that usually the information is provided mainly by the family members (like mother, grandmother, an aunt, or an elderly sister) followed by school teachers.

**Table 3:** Source and adequacy of knowledge about menstruation among participants who had received some information before menarche*.

	Young women	Adult women	Comments
	(*n* = 56)	(*n* = 67)	
**SOURCE OF INFORMATION (MULTIPLE RESPONSE)**			
Teacher	22 (39.3)	32 (47.8)	
Mother	20 (35.7)	12 (17.9)	
Other family members	10 (17.9)	12 (17.9)	
Other sources	06 (10.7)	26 (38.8)	
Physician	04 (7.1)	00 (0)	
**KNOWLEDGE RECEIVED REGARDING THE LINK BETWEEN**			

**MENSTRUATION AND FERTILITY**			
Yes	20 (35.7)	12 (18.5)	Chi square = 4.60, *p* = 0.032, significant
No	36 (64.3)	53 (81.5)	
**PERCEIVED ADEQUACY OF THE EXPLANATION OF MENSTRUATION**			
Adequate information received	13 (23.2)	05 (7.7)	Chi square = 4.56, *p* = 0.033, significant
Inadequate information received	43 (76.8)	60 (92.3)	

*Values are given as number (percentage).

Table 2 also shows that 70.8% young women faced physical complaints or health problems during menstruation. 65.6% adult women felt the same regarding their daughters. The details about the problems faced during menstruation by young women are presented in Table 4. The most common problem faced during menstruation was pain in abdomen (dysmenorrhea) followed by backache and body ache. A few complained about extensive or irregular bleeding. The two groups of women were quite similar in their views regarding types of menstruation problems. A higher proportion of the younger women said that they relied on self-treatment. On the other hand, the adult women thought the daughters consult their mother, mother-in-law, or significant others to a higher extent. Again physicians or gynecologist are rarely visited for consultation. Qualitative methods revealed that most of the girls do not face any problems in school and that the teachers were quite supportive. But in certain public schools where there were no special toilet facilities, the girls had to go home. Some girls mentioned that sanitary pads were available in some schools in case of emergency. The most common worries expressed during qualitative methods were the risk of staining while outside the home, missing the school, the concern about what is happening to the body, and the pain experienced during the menses. A few girls also mentioned that they worried a lot when their menses were delayed. Other health problems like skin rash, infections, etc. were not so common.

**Table 4:** The complaints faced by the participants and their approach during menstruation*.

	Young women	Adult women
	(*n* = 68)	(*n* = 63)
**PROBLEMS DURING MENSTRUATION (MULTIPLE RESPONSE)**		
Pain in abdomen (dysmenorrhea)	48 (70.6)	51 (81.0)
Backache	19 (27.9)	15 (23.8)
Body ache	16 (23.5)	06 (9.5)
Others (weakness, giddiness, etc.)	13 (19.1)	16 (25.4)
**APPROACH DURING PROBLEMS (MULTIPLE RESPONSE)**		
Self-treatment	34 (50.0)	21 (33.3)
Mother, mother in law or significant other	31 (45.6)	39 (61.9)
Doctors or gynecologist	07 (10.3)	10 (15.9)

*Values are given as number (percentage).

**Figure 1:**
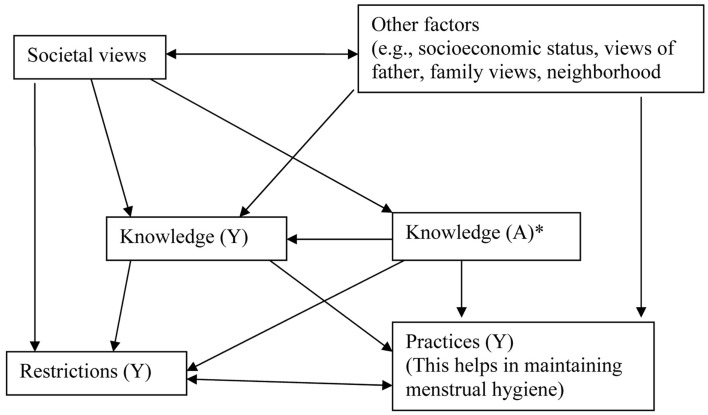
**The factors influencing the menstrual hygiene practices and restrictions among young women**. Y, young women; A, adult women. *Knowledge (A) among adult women is basically their own knowledge and what they know about knowledge and practices followed by young women.

Table 2 also shows that while 85.4% young women faced restrictions during menstruation, 88.5% adult women felt the same regarding their daughters. Majority of the participants have some kind of restrictions on them during the menstruation and most of these are religious restrictions (97.6%) rather than physical or social restrictions (10.8%). Few women (22.9%) said that the presence of a male family member during the menstrual periods was intimidating for young women. The qualitative results summarized in Figure 1 presents the factors influencing the menstrual hygiene practices and restrictions among young women. The figure also demonstrates inter-dependence of various factors. It was revealed that women cannot discuss openly at home about menstrual issues and they are considered unclean and untouchable during their menstrual periods. They are not allowed to carry out religious functions and not supposed to participate in the cooking during these periods. On the third/fourth day of the period, women are supposed to wash their hair. Few girls explicitly said they had been told how to maintain their hygiene during their periods. Girls were also advised to take a bath and wash their hair at the end of the period. A few girls had been told not to move around too much during the period and not to involve with boys. In spite of these restrictions, participants felt that they are acceptable to their family members during the menstrual period.

Sanitary napkins (either alone or along with reusable cloth) are used by the majority (74.5%) of the young women. But it is surprising to see that in urban area almost 25% participants are still using cloths. FGDs and KI interviews revealed that the most common advise given to the young women was that they should use sanitary pads/napkins and change it 2–4 times/day. However, quite a few girls were also advised to use cloth. Many girls seemed to start with cloths and move to sanitary napkins later on. Some girls had been told to use cloths when at home and to use sanitary pads when moving outside their home. On the other hand, cloths were said to be better when the bleeding was heavy since it could soak better than the sanitary pads. Health personnel recommend sanitary pads if the woman has economic resources. Otherwise according to them, cloths can be used given that they are carefully washed. During qualitative methods, it was discussed how do women dispose used sanitary protection material and how is this influenced by cultural rules. Girls were told by their mothers that cloths should be washed, dried, and reused. The majority of them also washed the used sanitary napkins and wrapped them in a newspaper or a plastic bag before disposal. The reasons for washing disposable pads are the fear of bad smell but also myths such as “*a snake coming to eat the blood stained pad putting the woman at risk of infertility or a curse*.”

Most of the girls said that they usually buy their sanitary pads themselves. Few said that someone else, most often the mother buys it for them. According to the girls, the mean cost for sanitary protection during a month was 40–45 INR with the range between 20 and 100 INR. For the girls who were still in school the mother had to provide the money. The cost was considered okay, although those who used cloth, which did not carry any costs, might have used this at least partly because of lack of money.

## Discussion

In our study, the mean age at menarche was 13.4 years, which is similar to another study conducted in urban slum of Delhi, India (6). It is expected that the girls are provided correct information about menstruation before its onset, i.e., menarche. In our findings, it is seen that either no or very poor information was provided to the adolescent girls before the onset of menstruation. A culture of silence surrounds menarche, an event which takes many young girls almost by surprise. The source of information was usually through school teachers and family relations especially mother, which is a good thing. But again it was seen that the information provided was not adequate enough. The inadequate knowledge of mother was passed on to these young girls.

These findings are similar to other studies in Delhi where most of the young girls were previously unaware that it would happen and the information they are given is sparse (6, 7). Similar findings are also seen from other parts of India like Rajasthan (8), Gujarat (9), Haryana (10), and Kerala (11). These studies in India also found that mothers, sisters, and friends are usually the major source of information (7–9, 12). Similar findings are also seen from studies in other parts of the world like Egypt (13), Pakistan (14), where it as seen that either inadequate or poor information was passed to the young girls through improper sources.

In our study, only 25% young women used reusable cloths (either alone or along with sanitary napkins). Obviously due to the poor socio economic conditions of these families, they cannot afford to purchase costly sanitary napkins. But it is essential that the reused cloths should be properly washed.

In a study from Rajasthan (8) and Delhi (7), the majority of the young girls were using and reusing old cloth, homemade napkins, and very few used cotton wool or sanitary napkins. Cloth is the cheapest material used for protection during menstruation. All kinds of old, ragged, and rejected clothes are kept by women for this and used by the majority of women in the slum and in rural areas (5, 6, 15). The main reasons for using homemade napkins were the inability to buy costly readymade sanitary napkins but also the lack of availability in rural areas (15).

Another important factor influencing their choice of sanitary protection methods was good absorbing capacity so there would not be any staining on the clothes. This question was, however, not elaborated in detail. Girls also need privacy and hygienic facilities at home as well as at school. Sanitary pads should be made available at schools for emergency use.

Limited knowledge can result in many unhygienic practices like reusing same cloth again and again without proper washing, ignoring health problems, trying to manage the problems faced during menstruation on their own, etc. These adolescent and young girls frequently face problems and restrictions during the menstruation and these problems are usually not tackled in a proper manner.

In a study in Nepal, it was seen that adolescent girls were not properly maintaining menstrual hygiene (16). The cloth used for menstruation should be clean otherwise it will become septic and form pus. Although during a study in Delhi (6), the women were taught to use clean cotton cloth and were aware of the consequences of using dirty cloth, observation revealed that old clothes were often kept in a dirty bundle to be used during the periods.

Washing the cloth is problematic since nobody especially men, should see any sign of menstruation. As a consequence, girls and women will have to use moisture and damp cloths. Some women think that the same cloth should not be used again since “it is a kind of disease” and all types of germs are discharged from inside (17). The concepts of the evil-eye and magic are strong especially in rural India. Thus, its disposal assumes a special significance in the daily lives for Indian women (5).

A study in Delhi (12) reported that among adolescent girls, dysmenorrhea was the most common problem followed by pre-menstrual syndrome. Our findings are almost same. This affects the daily routines of majority of the girls leading to absenteeism from school/college and work. Studies from out of India also reported similar findings. A study from Hong Kong showed that the prevalence of menstrual problems and dysmenorrhea increased with age but very few had sought medical care because of menstrual problems (18). A study in New Zealand showed many adolescent girls always experienced some pain during their menstruation and they stated that menstruation affected their daily activities (19). In Taiwan, a study among young girls aged 10–12 years reported that the girls experienced physical and emotional difficulties during menstruation (20).

Menstrual hygiene is one of the important risk factors for reproductive tract infections (21). Adolescent girls have been reported to suffer from various reproductive health problems associated with menstruation. Abdominal pain, bad odor of menstrual blood, burning during urination, and profuse discharge of menstrual blood were the most reported problems (8, 22). Women who use cloth are twice as likely to have bacterial vaginosis compared to women who use nothing during menstruation (15). Impact in view of reports of high levels of sexual activity, often at very young ages, and without protection, and the high risk for acquiring sexually transmitted diseases, the failure to adequately educate girls about their own anatomy and physiology has serious implications (23).

As per a study in Delhi, India shows that 92% were restricted in religious and social activities (8). In Gujarat, India, many families continue the custom of celebrating the first menarche and observing social restrictions (10). Studies thus show a rather harsh situation for girls and women who are considered as unclean (5, 8) and untouchable while menstruating and as a consequence kept away from normal activities of life by not leaving the home (6), presumably missing work and school, or being unable to attend places of worship (5), or to swim or exercise (24). The study in Ranchi in adolescent girls concludes that cultural and social practices regarding menstruation depend on girls’ education, attitude, family environment, culture, and belief (25). Many of the traditions and taboos surrounding menarche and menstruation are originally an outgrowth from religion (2) – a factor that is relatively stable across districts and local groups throughout India, as expressed by the influence religion continues to play on attitudes toward the phenomena (26).

## Conclusion

We conclude from our study that very few young girls between the age group 15 and 24 years did receive any information before the onset of menstruation. Among those who received some information, it was not adequate enough. The source of information was also not authentic. Both young and adult women agreed on this. Due to the inadequate knowledge, there were certain unhygienic practices followed by the young girls resulting in poor menstrual hygiene. It also leads to many unnecessary restrictions on young girls and they faced many health problems and complaints, which were either ignored or managed inappropriately. The role of the health sector was almost negligible in giving information to the management of health problems of these young girls.

This paper reemphasizes the important, urgent, and neglected need of providing correct knowledge to the entire community including adolescent and young girls. Correct knowledge will help them practice safe and hygienic menstrual practices and come out of traditional beliefs, misconceptions, and restrictions regarding menstruation. For this, the proper policies should be formulated and implemented, which can be part of overall health and community development policy. The physiology of the menstrual cycle, its connection to fertility, and the fact that menstruation is a normal process without any kinds of dirt should be part of family life/sexual education in schools. The health sector especially the public health system should play proactive role.

In order to eliminate needless limitations in relation to menstruation a number of changes are required in women’s social and reproductive health situation, community planning in relation to sanitation and disposal, and product development and marketing at a low cost. If the economic stature of India is changing as a whole, women’s situation with regard to menarche and menstruation may also change in the same direction.

The researchers feel that there were certain limitations associated with the study. Here, we could cover only urban lower-middle socio economic class population but they are not the poorest of the poor. Rural populations and ethnic minorities were not covered. The population was predominantly following Hindu religion. The views of boys and adult men could also not be investigated. It would be good to interview men/boys, pharmacists as well as religious leaders in order to understand these issues better. The study focus was quite broad and there was no focus on any particular specific issue.

The authors also felt that it was a good strategy using both qualitative and quantitative methods to collect data on such a sensitive topic. The FGD and the individual household interviews gave good information. The interviews with teachers and health workers gave answers that to some extent could be expected. The mothers might have given answers that are more related to what they hope they have done than what they actually have done. Researchers felt that the young girls should have been asked more about how they want information to be given and by whom but also where they want to buy sanitary pads and how much they think they can afford. There is definite need for a national level representative survey involving urban, rural, tribal population from different socio economic classes. Focused surveys especially on sensitive issues like affordability/financing, cultural issues, etc. will give important data.

## Conflict of Interest Statement

The authors declare that the research was conducted in the absence of any commercial or financial relationships that could be construed as a potential conflict of interest.
